# Development of a deep learning-based 1D convolutional neural network model for cross-species natural killer T cell identification using peripheral blood mononuclear cell single-cell RNA sequencing data

**DOI:** 10.14202/vetworld.2024.2846-2857

**Published:** 2024-12-18

**Authors:** Kaj Chokeshaiusaha, Thanida Sananmuang, Denis Puthier, Roongtham Kedkovid

**Affiliations:** 1 Department of Veterinary Science, Faculty of Veterinary Medicine, Rajamangala University of Technology, Tawan-OK, Chon Buri, Thailand; 2 Aix-Marseille Université, INSERM UMR 1090, TAGC, Marseille, France; 3 Department of Veterinary Medicine, Faculty of Veterinary Science, Chulalongkorn University, Bangkok, Thailand

**Keywords:** 1D convolutional neural network, K-means clustering, natural killer T cell, peripheral blood mononuclear cells, single-cell RNA sequencing

## Abstract

**Background and Aim:**

Natural killer T (NKT) cells exhibit the traits of both T and NK cells. Although their roles have been well studied in humans and mice, limited knowledge is available regarding their roles in dogs and pigs, which serve as models for human immunology. Single-cell RNA sequencing (scRNA-Seq) can elucidate NKT cell functions. However, identifying cells in mixed populations, like peripheral blood mononuclear cells (PBMCs) is challenging using this technique. This study presented the application of one-dimensional convolutional neural network (1DCNN) for the identification of NKT cells within scRNA-seq data derived from PBMCs.

**Materials and Methods:**

We used human scRNA-Seq data to train a 1DCNN model for cross-species identification of NKT cells in canine and porcine PBMC datasets. K-means clustering was used to isolate human NKT cells for training the 1DCNN model. The trained model predicted NKT cell subpopulations in PBMCs from all species. We performed Differential gene expression and Gene Ontology (GO) enrichment analyses to assess shared gene functions across species.

**Results:**

We successfully trained the 1DCNN model on human scRNA-Seq data, achieving 99.3% accuracy, and successfully identified NKT cell candidates in human, canine, and porcine PBMC datasets using the model. Across species, these NKT cells shared 344 genes with significantly elevated expression (FDR ≤ 0.001). GO term enrichment analyses confirmed the association of these genes with the immunoactivity of NKT cells.

**Conclusion:**

This study developed a 1DCNN model for cross-species NKT cell identification and identified conserved immune function genes. The approach has broad implications for identifying other cell types in comparative immunology, and future studies are needed to validate these findings.

## Introduction

Natural killer T (NKT) cells are heterogeneous immune cells that have the features of both conventional T and natural killer (NK) cells. NKT cells can fine-tune immune responses toward either inflammation or tolerance through the action of various cytokines, resulting in their potential roles in anti-tumor activity, autoimmune disease regulation, and even infection [1, 2]. The development and function of NKT cells have been intensively studied in mice and humans [1]. However, accumulating knowledge from studies has shown the importance of NKT cells in several mammalian species, including canine and porcine species, as well as well-recognized companion and livestock animal models for human medicine [3, 4]. Despite their well-established significance in human immunology, the nature of NKT cells in canine and porcine species remains largely enigmatic. Further exploration is essential for advancing our understanding of animal immunology and improving veterinary medicine.

The identification of NKT cells typically relies on the detection of surface molecules such as CD3 and Killer Cell Lectin-Like Receptor B1 (KLRB1) (CD161) via flow cytometry [1–4]. Although this method allows for the direct measurement of robust NKT cell phenotypes, it offers limited insight into the underlying changes in their gene expression. In contrast, single-cell RNA sequencing (scRNA-Seq) provides a comprehensive profile of all genes expressed in individual cells, including those encoding surface markers and other functional molecules. This approach effectively captures cellular heterogeneity, facilitating the identification and study of different NKT cell subpopulations [2, 5, 6]. However, gene expression does not consistently correlate with protein abundance [7], including that of surface marker genes, which necessitates NKT cell sorting prior to scRNA-Seq analysis [2, 5, 6]. This prerequisite significantly restricts the study of NKT cells from mixed immune cell environments, such as peripheral blood mononuclear cells (PBMCs).

The discrepancy between gene expression and expressed protein levels, coupled with the inherently noisy and imperfect nature of scRNA-Seq data due to the low starting amount of mRNA copies per cell, presents significant challenges for cell identification in mixed cell population data [8, 9]. Shorter genes are more likely to be dropped out during the amplification step, introducing further bias and resulting in inconsistent gene expression patterns within the same cell population. This inconsistency complicates the identification of cells of interest. In this context, rapidly emerging animal research deep-learning models offer promising solutions [10–12]. Models such as 1-Dimensional Convolutional Neural Networks (1DCNN) excel at automatically learning relevant features directly from the data and identifying patterns within sequential data [13]. Consequently, 1DCNNs should provide a productive approach for identifying NKT cell populations across species by recognizing similar expression patterns of universal marker gene expressions. Since this model could identify candidate NKT cells based on marker gene expression patterns, it was possible to use the model for mixed cell populations like PBMCs.

This study pioneered the application of 1DCNN modeling to cross-species NKT cell identification. Using the unique expression patterns of selected surface marker genes, we trained the model on human NKT cell datasets. The trained model was then successfully applied to the canine and porcine PBMC datasets, enabling the identification of potential peripheral NKT cells across these species. This novel approach not only introduced an alternative method for identifying canine and porcine NKT cell populations but also demonstrated the broader applications of deep learning in comparative immunology.

## Materials and Methods

### Ethical approval

The current study used publicly available secondary data from the NCBI SRA website (https://www.ncbi.nlm.nih.gov/sra). This research did not involve the use of animals or humans, and no animals or individuals were identifiable in this publication.

### Study period and location

The study was conducted from December 2023 to October 2024 at the Department of Veterinary Science, Faculty of Veterinary Medicine, Rajamangala University of Technology Tawan-OK, Chonburi, Thailand.

### Sample datasets

This study used scRNA-Seq datasets of unstimulated NKT cells [2] derived from a healthy human donor as references for semi-supervised clustering of human PBMC datasets [14]. Additionally, we used datasets from canine PBMCs of healthy dogs [15] and porcine PBMCs [16] to identify potential cross-species NKT cell candidates (Table-1). The references for each species showed specific protocol variations during data preparation.

**Table-1:** Single-cell RNA-sequencing datasets.

Cell type	Species	Dataset	Description
NKT cells	Human	SRR8724694	NKT cells isolated from unstimulated PBMCs
	Human	SRR8724695	NKT cells isolated from unstimulated PBMCs
	Human	SRR8724696	NKT cells isolated from unstimulated PBMCs
PBMCs	Human	SRR24688956	PBMCs isolated from a healthy human
	Human	SRR24688957	PBMCs isolated from a healthy human
	Human	SRR24688958	PBMCs isolated from a healthy human
	Human	SRR24688959	PBMCs isolated from a healthy human
	Human	SRR24688960	PBMCs isolated from a healthy human
	Human	SRR24688961	PBMCs isolated from a healthy human
	Dog	SRR23525329	PBMCs isolated from a healthy dog
	Dog	SRR23525330	PBMCs isolated from a healthy dog
	Dog	SRR23525331	PBMCs isolated from a healthy dog
	Dog	SRR23525333	PBMCs isolated from a healthy dog
	Pig	ERR5523708	PBMCs isolated from a healthy pig
	Pig	ERR5523709	PBMCs isolated from a healthy pig
	Pig	ERR5523711	PBMCs isolated from a healthy pig
	Pig	ERR5523710	PBMCs isolated from a healthy pig
	Pig	ERR5523712	PBMCs isolated from a healthy pig
	Pig	ERR5523713	PBMCs isolated from a healthy pig
	Pig	ERR5523714	PBMCs isolated from a healthy pig

NKT=Natural killer T, PBMC=Peripheral blood mononuclear cell

### Experimental design

**Figure-1:**
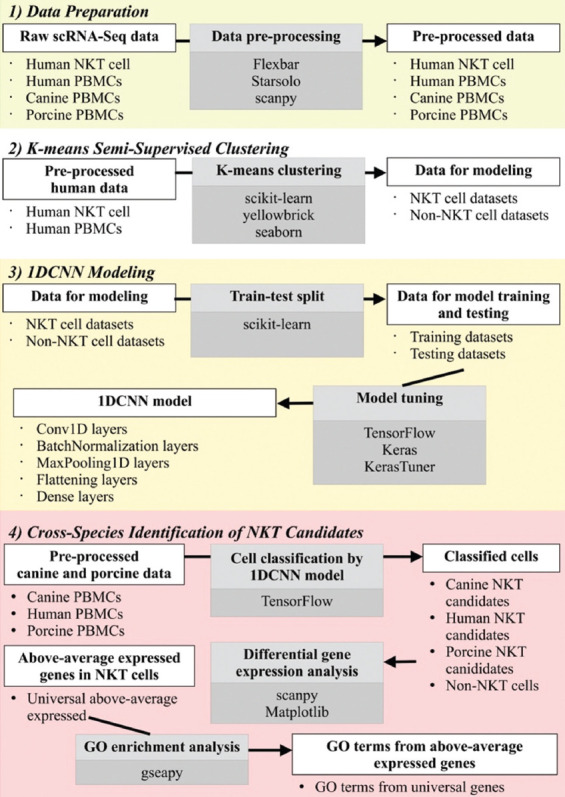
The workflow comprised four parts: (1) Data preparation, (2) K-means semi-supervised clustering, (3) 1DCNN modeling, and (4) Cross-species identification of NKT candidates. Each part involved specific analytical processes using different data types as in-puts and outputs. The white rectangles indicate the data types used as inputs or acquired as outputs of each process. In contrast, gray rectangles represent each analytical process along with the names of the used Python/R packages. The Materials and Methods section compre-hensively covers all analytical procedures. 1DCNN=1D Convolutional Neural Network, NKT=Natural killer T.

The experiment comprised four parts: (1) Data Preparation, (2) K-means semi-supervised clustering, (3) 1DCNN modeling, and (4) Cross-Species Identification of NKT Candidates (Figure-1). In the “Data Preparation” section, we pre-processed all scRNA-Seq datasets and transformed them into gene expression profiles of each cell type. The “K-means Semi-Supervised Clustering” section utilized the prepared gene expression profiles of isolated human NKT cells to identify candidate human NKT cell populations in PBMCs and subsequently used all expression profiles to train the 1DCNN model in the “1DCNN Modeling” section. The trained model was subsequently applied to human, canine, and porcine PBMC datasets in the “Cross-Species Identification of NKT Candidates” section to identify NKT cell candidates across species. These candidates were then characterized based on their elevated expression of orthologous genes.

#### Part 1: Data preparation

The preprocessing of raw scRNA-Seq datasets followed previously described procedures by Sananmuang *et al*.[17] and Zhou *et al*. [18]. We used the”Flexbar” program [19] to remove contaminated adapter sequences and discard sequences with a mean Phred score below 30. Next, we employed STARsolo software [20] to align the filtered sequences to the respective genome sequences for each species: GRCh38 for humans, CanFam3.1 for dogs, and Sscrofa11. for pigs (http://www.ensembl.org). We excluded low-quality cells with a total count of ≤10,000 and detection of ≤300 genes per cell while retaining only those with a mitochondrial read fraction below 25%. Additionally, the included cells were required to have at least five counts for at least one surface marker gene (Table-2). We used the”scanpy” toolkit in Python [21] for these filtering procedures, resulting in high-quality pre-processed cell data for further analysis.

**Table-2:** Surface marker genes.

Gene name	Encoded protein	Expressed cells
CD3D	CD3 Delta Subunit of the T-Cell Receptor Complex	NK, NKT, and T cells
CD3G	CD3 Gamma Subunit of the T-Cell Receptor Complex	NK, NKT, and T cells
CD3E	CD3 Epsilon Subunit of the T-Cell Receptor Complex	NK, NKT, and T cells
KLRB1	Killer Cell Lectin-Like Receptor B1	NK and NKT cells
FCGR3A	Fc Gamma Receptor IIIa	NK and NKT cells
NCAM1	Neural Cell Adhesion Molecule 1	NK and NKT cells
CD19	CD19 Molecule	B cells
SDC1	Syndecan 1	Plasma cells
CD14	CD14 Molecule	Monocytes

NK=Natural killer, NKT=Natural killer T

#### Part 2: K-means semi-supervised clustering

This section describes the methodology used to identify clusters of human NKT cells in PBMCs by applying K-means clustering as a semi-supervised method in Python, utilizing the “scikit-learn” and “yellowbrick” libraries [22]. To address overfitting in the subsequently trained model, we included only nine surface marker genes (Table-2) in this clustering process. To compare relative expression levels, we performed counts per million normalization and Z-score normalization within a sample of surface marker genes. With the known NKT cells, the concatenated expression data of human NKT cells and PBMCs were analyzed by K-means clustering. With optimal cluster numbers determined by the Elbow plot of the distortion scores, the cell cluster with the largest number of NKT cells would be a candidate NKT cell dataset for further 1DCNN model training. The other cell clusters are the non-NKT cell datasets for training. We used “seaborn” library for plotting heatmaps of all cell clusters.

#### Part 3: 1DCNN modeling

A 1DCNN analyzes sequential data. The model scans the data to identify patterns or relationships between features in the sequence [23]. In the present study, the features were the normalized expression values of target surface marker genes (Table-2). A 1DCNN comprises four major components, each containing different structural layers [13]:

Convolutional component

This component takes the sequential expression values of target surface marker genes as input. The proposed method used multiple filters that slid along the data to identify specific patterns or motifs. Each filter had a certain width (kernel size) to define the number of data points it considered at a time. Each filter also learned a set of weights and biases to emphasize crucial features and downplay irrelevant information. Summing the results of these processes, the network generates a feature map that captures the presence and strength of the features detected by each filter in different parts of the data. The proposed component uses Conv1D layers with filters and kernels to extract features from segments of the sequence data by identifying recurring patterns.

Normalization component

This component included BatchNormalization layers, which played a crucial role by standardizing the data across training data batches, leading to smoother and more efficient training.

Pooling component

The pooling component reduced the data and focused on the most crucial features. The proposed method used MaxPooling1D layers to reduce the dimensionality of the data by selecting the most significant features within a defined window size (pool size). This layer acted like a downsampling tool, keeping the maximum value within each window of data points, diminishing the data’s dimensionality while summarizing the key points.

Fully connected component

Finally, the fully connected layers integrated information from all segments to make a final decision. After flattening the data into a one-dimensional vector by the flattening layers, the dense layers integrated the information extracted from all segments by the convolutional component and performed the classification to determine whether the input cell was an NKT cell. The layers used specific activation functions to decide whether a neuron should be activated.

We designed 1DCNN models to classify NKT cells from non-NKT cell populations based on surface gene marker expression using the TensorFlow, Keras, and KerasTuner libraries [24] in Python. Leveraging human NK cell and non-NK cell datasets previously prepared by the K-means clustering step, we split the data into training and testing datasets with a 20% test size using the train_test_split function of the scikit-learn library. The hyperparameters of the 1DCNN model were tuned using the KerasTuner library. The designed models were trained on the prepared training datasets with a validation split of 30% to monitor their performance during training. The trained models incorporated the early stopping regularization and the categorical cross-entropy loss function during model compilation with the Adam optimizer as an algorithm for parameter adjustment (Learning rate = 0.001). With the test datasets, the model performances were evaluated and compared using accuracy, precision, recall, and F1 scores. The predicted probability values for the NKT and all non-NKT cell classes provided the final predictions for each cell in the test datasets. We selected the best-performing model for cross-species NKT cell identification. We calculated accuracy, precision, recall, and F1 scores using equations 1, 2, and 3, respectively.


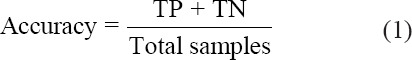



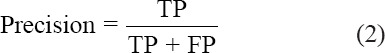



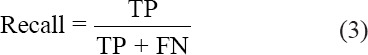



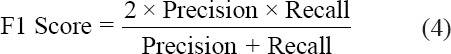


Where,

TP (True positive): Represented the number of cells correctly classified in the classes.

TN (True negative): Represented the number of cells correctly not classified in other classes.

FP (False positive): Represented the number of cells incorrectly classified in other classes.

FN (False negative): Represented the number of cells incorrectly not classified in their classes.

#### Part 4: Cross-species identification of NKT candidates

The optimal 1DCNN model was used to predict class probabilities for each cell sample in the canine, human, and porcine PBMC data (threshold = 0.95). We designated each cell sample to the class with the highest predicted probability (NKT cell class or other non-NKT cell classes). We created heatmaps using the seaborne library to visualize the expression patterns of cell marker genes across different cell clusters. For the concatenated PBMC data, we conducted differential gene expression analysis between NKT and non-NKT cells across all species using the modified t-test method of the scanpy toolkit in Python (false discovery rate - ≤ 0.001 and Log_2_Fold-change ≥2). After identifying genes with above-average expression in candidate NKT cells, we employed the Matplotlib_venn library in Python to generate a Venn diagram, allowing us to visualize the overlap of these genes among the species. The genes shared by all three species were then subjected to Gene Ontology (GO) enrichment analysis using the GSEApy library in Python to identify significantly overrepresented biological processes, molecular functions, or cellular components within this gene set.

## Results

### Surface marker gene expression patterns in human NKT cells

**Figure-2:**
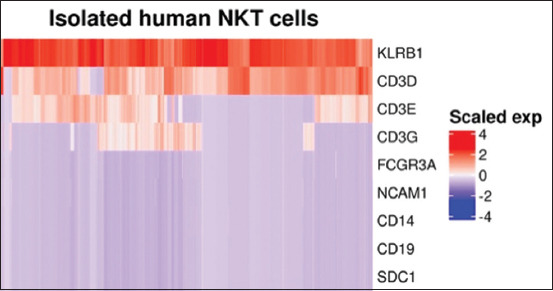
Surface marker gene expression patterns of isolated human NKT cells. The heatmap shows the scaled expression levels of surface marker genes in the isolated human NKT cells. NKT=Natural killer T.

We successfully prepared scRNA-seq data for subsequent analyses. However, excluding cells with insufficient surface marker gene expression led to the removal of a significant number of cells from the PBMC datasets. After data preparation, the total number of cells obtained from humans, canine, and porcine PBMCs was 4147, 2230, and 1583, respectively. The total number of human NKT cells was 5120 after preparation. Examination of the isolated human NKT cell data revealed a unique surface marker gene expression pattern characterized by rather consistent KLRB1 expression across the cells despite the varying expression levels of CD3D, CD3E, and CD3G (Figure-2).

### Human NKT cell cluster identified via K-means clustering

**Figure-3:**
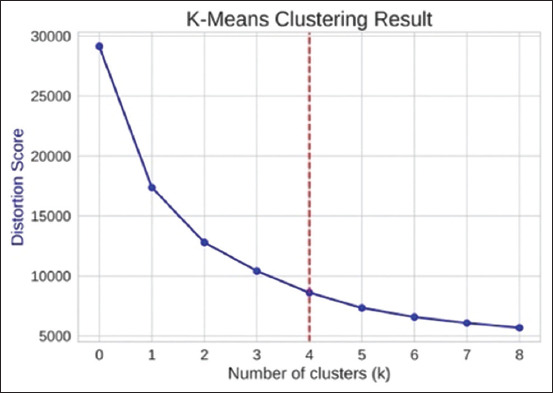
Elbow plot. The X and Y axes of the plot represent the number of clusters (k) and the score metric (the distortion score), respectively. As the number of clusters increased, the score decreased until k = 5, beyond which the reduction in score became insignificant (the el-bow point). Since adding more clusters beyond this point did not significantly improve the explanation of the data’s variance, the optimal number of clusters would be the value just be-fore the elbow point, which was k=4 in this plot.

**Figure-4:**
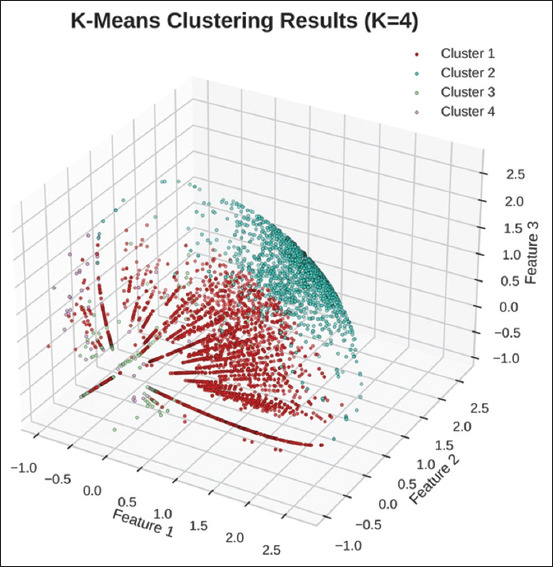
K-means clustering results visualized using t-distributed stochastic neighbor em-bedding (t-SNE). The figure displays the results of K-means clustering of human cells with four clusters (K = 4), which were visualized using t-SNE dimensionality reduction. Each point represents a cell point colored according to its assigned cluster.

Using the concatenated expression data of human NKT cells and PBMCs, the Elbow plot identified 4 clusters (k = 4) as the optimal number for K-means clustering (Figure-3). The first cell cluster containing the highest number of known NKT cells (5074 cells) was indicated as the candidate NKT cell data for further 1DCNN model training. The other cell clusters were classified as non-NKT cell datasets (Figure-4).

### Optimization of the 1DCNN architecture and parameters

**Figure-5:**
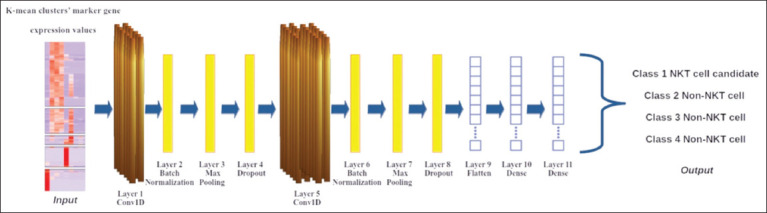
Architecture of best performing 1DCNN model. The figure illustrates a 1DCNN architecture based on marker gene expression profiles. The input to the network is a matrix of gene expression values for each cell cluster. The CNN architecture comprised multiple convo-lutional layers, batch normalization layers, max pooling layers, dropout layers, and fully con-nected layers. The output layer predicted the class of each cell, with four possible classes: Class 1 NKT cell candidate, Class 2 Non-NKT cell, Class 3 Non-NKT cell, and Class 4 Non-NKT cell. 1DCNN=1D Convolutional Neural Network, NKT=Natural killer T.

Using the expression values of surface marker genes of human NKT and non-NKT cell clusters as input features, we constructed various 1DCNN architectures using KerasTuner. We explored various model architectures and ultimately selected the optimized architecture because it consistently outperformed the other models in terms of performance metric scores. The best-performing model, trained on the dataset, achieved an accuracy of 99.3%, with precision, recall, and F1 scores of 91.5%, 99.9%, and 95.5%, respectively (Table-3). We illustrate the finalized model structure (Figure-5) and summarize its details in Table-4.

**Table-3:** Performance metrics of the optimized 1DCNN model.

Metrics	Values
Accuracy	0.993
Precision	0.915
Recall	0.999
F1-score	0.955

1DCNN=1D Convolutional Neural Network

**Table-4:** Summary of the 1DCNN model architecture.

Model type	1DCNN
Architecture	Layer 1 - Conv1D (32 filters, kernel size 2): Features are extracted from the input data using 32 filters of size 2 with the rectified linear unit activation function.
	Layer 2 - BatchNormalization: This layer is normalized to improve training stability.
	Layer 3 - MaxPooling1D (pool size 2): The dimensionality of the data is reduced by taking the maximum value from every window of size 2
	Layer 4 - Dropout (0.2): Randomly drop 20% of the activations during training to prevent overfitting.
	Layer 5 - Conv1D (64 filters, kernel size 2): This layer extracted higher-level features with 64 filters similar to the first Conv1D layer.
	Layer 6: BatchNormalization
	Layer 7 - MaxPooling1D (pool size 2): Similar to the first MaxPooling1D layer.
	Layer 8 - Dropout (0.2): Similar to the first Dropout layer.
	Layer 9 - Flatten: Reshapes the data to a one-dimensional vector.
	Layer 10 - Dense (128 units, Rectified Linear Unit—ReLU activation): The first fully-connected layer with 128 neurons and ReLU activation.
	Layer 11 - Dense (4 units, sigmoid activation): Output layer with 4 units and sigmoid activation for predicting probabilities of belonging to 4 different classes (1 NKT cell class and 3 non-NKT cell classes)
Model compilation	Loss: Categorical cross-entropy Optimizer: Adam optimizer with a learning rate of 0.001 Metrics (to monitor classification performance): Accuracy, Precision, Recall, and F1 score

1DCNN=1D Convolutional Neural Network, NKT=Natural killer T

### NKT cell candidates identified using the 1DCNN model across species

**Figure-6:**
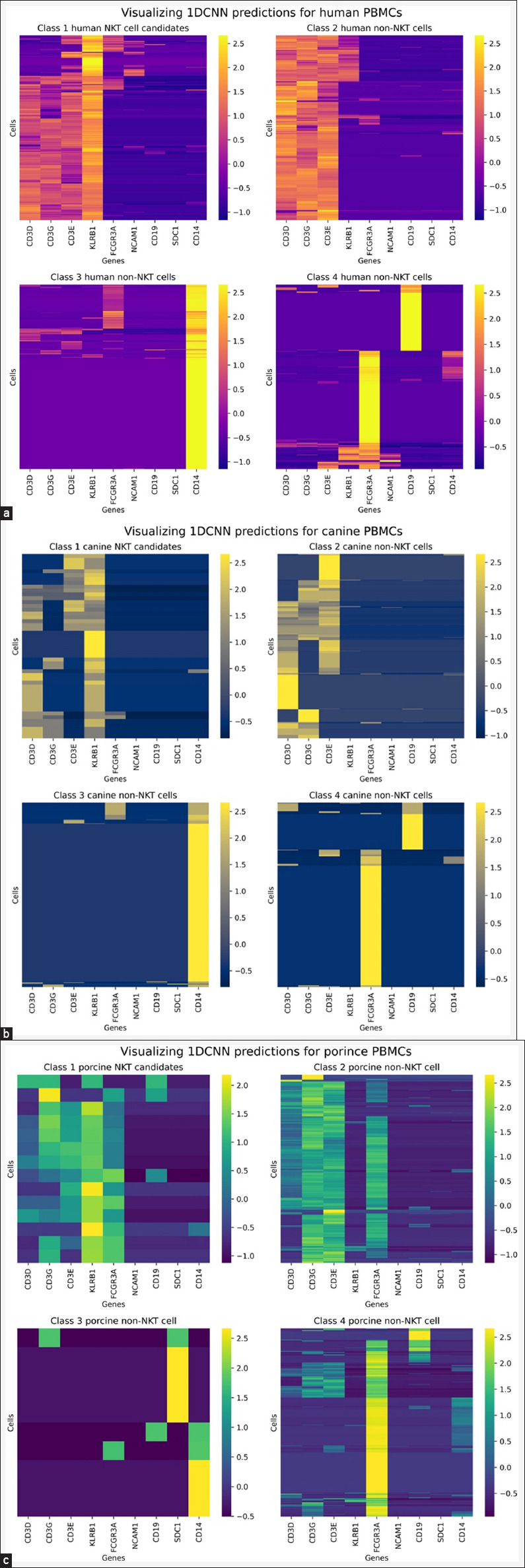
Visualization of 1DCNN predictions across species. The heatmap depicts the expression of surface marker genes in (a) human and (b) canine. (c) porcine PBMCs, as classified by the cross-species 1DCNN model. The NKT cell candidates were consistently classified as Class 1 in all species. 1DCNN= 1D Convolutional Neural Network, NKT=Natural killer T, PBMC=Peripheral blood mononuclear cell.

**Figure-7:**
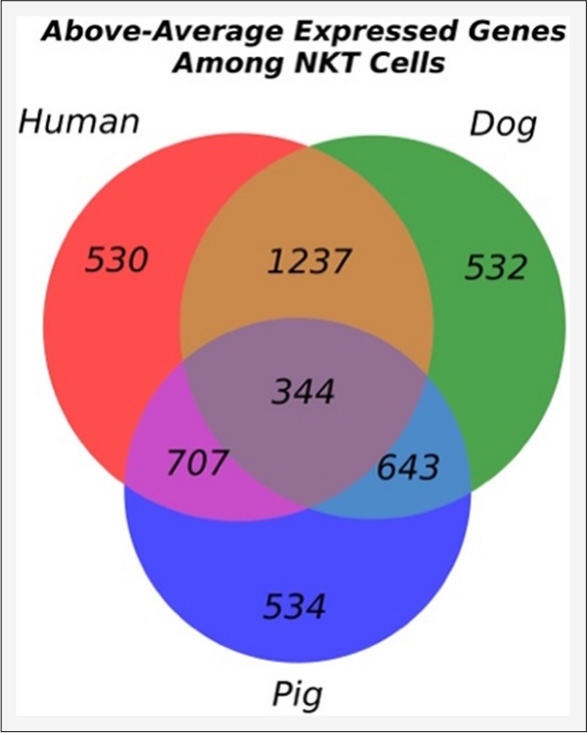
Venn diagram illustrating the overlap of genes expressed above average in humans, dog, and pig NKT cells. The genes uniquely expressed in humans, canine, and porcine NKT cells were 539, 532, and 534, respectively. The genes expressed in both human and canine NKT cells were 1237. The genes expressed in both human and porcine NKT cells were 707. The genes expressed in dog and pig NKT cells were 643, and those expressed in all three spe-cies were 344. NKT=Natural killer T.

**Figure-8:**
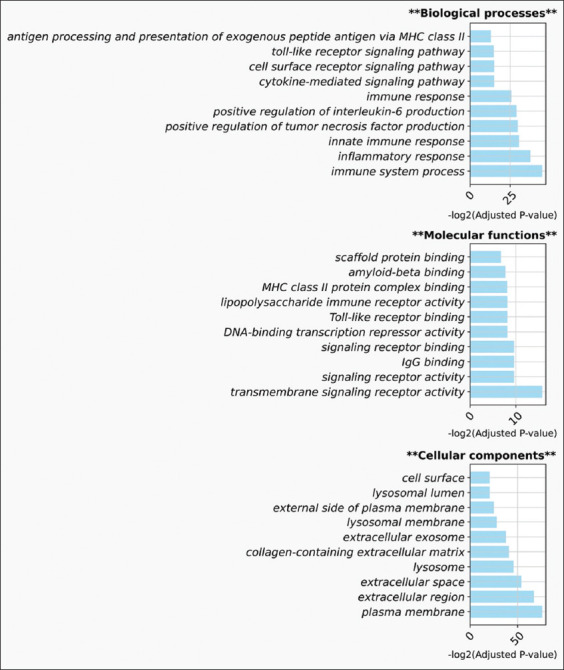
GO enrichment analysis of genes with above-average expression in humans, canine, and porcine NKT cells. We present the top 10 significant terms according to their categories: Biological Processes, Molecular Functions, and Cellular Components. GO=Gene ontology, NKT=Natural killer T.

Using the best-performing 1DCNN model, we predicted the class probabilities of cells in the PBMC datasets for all species. By threshold = 0.95, the model identified 360, 160, and 60 NKT cell candidates in humans, canine, and porcine PBMCs. These percentages accounted for 8.76%, 10.17%, and 2.77% of the cells in the PBMCs, respectively. Notably, we observed similar expression patterns of cell marker genes among the NKT cell candidates across the three species (Class 1 in Figures-6a–c). Differential gene expression analysis showed variation in the number of genes with high expression (p ≤ 0.001, Log2 fold-change ≥2) among NKT cell candidates from different species. Human and canine NKT cells shared 1237 genes, whereas human and porcine NKT cells shared 707 genes. Only 344 genes were common across all three species (Figure-7). GO term enrichment analysis further indicated that many of these shared genes were associated with immune signaling, immune activity, and cellular components, such as the cell membrane and transport vesicles (Figure-8).

## Discussion

In the scRNA-Seq analysis of PBMCs, the inherent variability in gene expression patterns within the same cell population presents a significant challenge for identifying minor cell populations, such as NKT cells. This challenge is even more complex in cross-species studies, where cell populations of the same type may exhibit distinct characteristics across species, leading to different cell subpopulations across mammalian species [2–4]. For the first time, this study introduced a novel method for cross-species NKT cell identification in PBMCs using a 1DCNN model on their scRNA-Seq expression data. Using the expression patterns of selected surface marker genes as features, we successfully trained the 1DCNN model using isolated human NKT cell and PBMC data, achieving high accuracy. When applied to canine and porcine PBMC data, the model accurately predicted NKT cell subpopulations with similar marker gene expression patterns. These subpopulations also shared significant expression of specific immune function-related genes among human, canine, and porcine species.

This study included expression data from isolated human NKT cells and NKT cells within human PBMCs obtained via semi-supervised K-means clustering (Figure-4) to train the 1DCNN model. The expression patterns of cell marker genes in human NKT cells aligned well with previously reported surface protein expression in peripheral NKT cells by Liu *et al*. [25]. These characteristics included notably high expression levels of KLRB1 and CD3-associated genes, specifically CD3D, CD3E, and CD3G (Figure-2). Despite the prominence of these expression levels, their considerable variability was noticeable among human NKT cells (Figure-2). This variability underscored the value of training the 1DCNN to capture such distinctive patterns because the model could effectively identify local patterns and their dependencies [13, 23]. Our K-means clustering analysis revealed four distinct cell clusters within human PBMCs, suggesting four biologically relevant cell subgroups with unique characteristics (Figure-4). These differences suggest that a single category of non-NKT cells might not adequately capture the complexity of PBMCs. Instead, a 4-class model would likely offer a more nuanced understanding of the cellular landscape, leading to more precise biological interpretations, especially for subsequent differential gene expression analyses. For the subsequent 1DCNN training, we utilized the expression data of all four cell clusters.

Using the optimized model (Figure-5), we predicted the class probabilities of each cell sample across all species. To ensure reliable identification of NKT cell candidates, we applied strict criteria, including adequate expression of surface marker genes in PBMCs and setting a high prediction threshold of 0.95 for the 1DCNN model. This stringent approach likely contributed to the reduced number of pre-processed cells and consequently the limited number of identified NKT cells across all species. All cells classified as Class 1 NKT cell candidates across the three species exhibited strong similarities in the expression of KLRB1 and CD3-associated genes. In contrast, cells in Classes 2 and 3 consistently exhibited high expression of CD3-associated genes and CD14, indicating T cell and monocyte characteristics. Notably, porcine Class 3 samples also exhibited significant SDC1 expression, suggesting the contribution of plasma cells. Meanwhile, Class 4 cells represented a mixed population of B cells and NK cells, characterized by prominent CD19 and FCGR3A expressions (Figure-6).

Although direct comparative studies are lacking, earlier attempts to characterize human, canine, and porcine NKT cells have suggested significant variations in cell characteristics and subpopulations among these species [3, 4, 15, 25]. These variations suggested species-specific differences in gene expression, although some conserved features still reflect core NKT cell characteristics. The differing numbers of NKT cell candidates identified by 1DCNN across species and the limited overlap in differentially expressed genes illustrated in this study further supported these notifications (Figure-7). Another contributing factor to this outcome was the diversity of cell populations within the PBMCs of each species, which could affect the mean expression levels of genes. Because this study identified NKT cell feature genes based on their higher expression relative to mean levels, such variation could also influence the test’s sensitivity. Future scRNA-Seq studies of isolated NKT cells across all species are needed to confirm these outcomes.

Despite the previously described limitations, our analysis revealed a striking pattern of gene expression similarity between human and dog NKT cells throughout the large number of shared orthologous genes expressed above the mean values (1237 genes in Figure-7). This suggested a close evolutionary relationship or shared functional requirements between the two species. While pigs also shared a common ancestor with humans, the lesser degree of overlapped genes observed (707 genes in Figure-7) in our study may indicate distinct evolutionary trajectories or specialized adaptations in their NKT cell biology. These results underscore the importance of comparative genomics for understanding the functional conservation and divergence of immune cell subsets across species.

The GO term enrichment analysis revealed several conserved genes with elevated expression in NKT cell functions across the three species. Many significant GO terms were directly related to immune activity through transmembrane signaling and antigen recognition by NKT cells (Figure-8). These mechanisms are essential for NKT cells, enabling their interaction with their environment and allowing them to play pivotal roles in innate and adaptive immunity [1, 26]. Other significant GO terms supported these crucial functions, such as antigen recognition, inflammation, and cytokine responses (Figure-8). Notably, the identification of highly expressed, conserved genes among NKT cells across species indicated that these genes likely played fundamental roles in NKT cell biology and function and were conserved throughout evolution.

## Conclusion

This study successfully introduced a novel approach in veterinary research by applying a 1DCNN model to identify cross-species NKT cells in PBMCs using scRNA-Seq expression data. By leveraging the expression patterns of selected surface marker genes, the model achieved high accuracy in identifying NKT cell subpopulations in humans, canine, and porcine PBMCs. The results suggest that certain NKT cell function-related genes are conserved across species, highlighting their fundamental roles in NKT cell biology and function. This innovative application of deep learning to cross-species analysis advanced our understanding of NKT cells and provided new avenues for future veterinary research. Further studies should focus on direct scRNA-Seq comparative analyses of isolated NKT cells in humans, canines, and porcines to confirm and refine these findings.

### Data Availability

We provided a list of orthologous genes that NKT cells expressed above average expression values shared among human, canine, and porcine species in TXT format. Other data may be made available upon request.

## Authors’ Contributions

KC, TS, and RK: Study design, collected the datasets, and analyzed the data. TS and DP: Refined the study design and objective. DP: Performed technical coding correction and hardware maintenance. KC and RK: Drafted, reviewed, and revised the manuscript. All authors have read and approved the final manuscript.

## References

[1] Brennan P.J, Brigl M, Brenner M.B (2013). Invariant natural killer T cells:An innate activation scheme linked to diverse effector functions. Nat. Rev. Immunol.

[2] Zhou L, Adrianto I, Wang J, Wu X, Datta I, Mi Q.S (2020). Single-cell RNA-seq analysis uncovers distinct functional human NKT cell sub-populations in peripheral blood. Front. Cell. Dev. Biol.

[3] Gingrich A.A, Modiano J.F, Canter R.J (2019). Characterization and potential applications of dog natural killer cells in cancer immunotherapy. J. Clin. Med.

[4] Schäfer A, Hühr J, Schwaiger T, Dorhoi A, Mettenleiter T.C, Blome S, Schröder C, Blohm U (2019). Porcine invariant natural killer T cells:Functional profiling and dynamics in steady state and viral infections. Front. Immunol.

[5] Wang J, Adrianto I, Subedi K, Liu T, Wu X, Yi Q, Loveless I, Yin C, Datta I, Sant'Angelo D.B, Kronenberg M, Zhou L, Mi Q.S (2023). Integrative scATAC-seq and scRNA-seq analyses map thymic iNKT cell development and identify Cbf for its commitment. Cell. Discov.

[6] Rotolo A, Whelan E.C, Atherton M.J, Kulikovskaya I, Jarocha D, Fraietta J.A, Kim M.M, Diffenderfer E.S, Cengel K.A, Piviani M, Radaelli E, Duran-Struuck R, Mason N.J (2023). Unedited allogeneic iNKT cells show extended persistence in MHC-mismatched canine recipients. Cell. Rep. Med.

[7] Li J, Zhang Y, Yang C, Rong R (2020). Discrepant mRNA and protein expression in immune cells. Curr. Genomics.

[8] Jia C, Hu Y, Kelly D, Kim J, Li M, Zhang N.R (2017). Accounting for technical noise in differential expression analysis of single-cell RNA sequencing data. Nucleic Acids Res.

[9] Kim J.K, Kolodziejczyk A.A, Ilicic T, Teichmann S.A, Marioni J.C (2015). Characterizing noise structure in single-cell RNA-seq distinguishes genuine from technical stochastic allelic expression. Nat. Commun.

[10] Erfanian N, Heydari A.A, Feriz A.M, Iañez P, Derakhshani A, Ghasemigol M, Farahpour M, Razavi S.M, Nasseri S, Safarpour H, Sahebkar A (2023). Deep learning applications in single-cell genomics and transcriptomics data analysis. Biomed. Pharmacother.

[11] Arisdakessian C, Poirion O, Yunits B, Zhu X, Garmire L.X (2019). DeepImpute:An accurate, fast, and scalable deep neural network method to impute single-cell RNA-seq data. Genome Biol.

[12] Sananmuang T, Mankong K, Chokeshaiusaha K (2024). Multilayer perceptron and support vector regression models for feline parturition date prediction. Heliyon.

[13] Zhou F.Y, Jin L, Dong J (2017). Review of convolutional neural network. Jisuanji Xuebao/Chin. J. Comput.

[14] Wu X.H, He Y.Y, Chen Z.R, He Z.Y, Yan Y, He Y, Wang G.M, Dong Y, Yang Y, Sun Y.M, Ren Y.H, Zhao Q.Y, Yang X.D, Wang L.Y, Fu C.J, He M, Zhang S.J, Fu J.F, Liu H, Jing Z.C (2023). Single-cell analysis of peripheral blood from high-altitude pulmonary hypertension patients identifies a distinct monocyte phenotype. Nat. Commun.

[15] Ammons D.T, Harris R.A, Hopkins L.S, Kurihara J, Weishaar K, Dow S (2023). A single-cell RNA sequencing atlas of circulating leukocytes from healthy and osteosarcoma affected dogs. Front. Immunol.

[16] Herrera-Uribe J, Wiarda J.E, Sivasankaran S.K, Daharsh L, Liu H, Byrne K.A, Smith T.P.L, Lunney J.K, Loving C.L, Tuggle C.K (2021). Reference transcriptomes of porcine peripheral immune cells created through bulk and single-cell RNA sequencing. Front. Genet.

[17] Sananmuang T, Puthier D, Nguyen C, Chokeshaiusaha K (2023). Differential transcript usage across mammalian oocytes at the germinal vesicle and metaphase II stages. Theriogenology.

[18] Kuendee N, Puthier D, Nguyen C, Chokeshaiusaha K (2021). Activation-induced cell death of NK cells in canine atopic dermatitis revealed by Droplet-Sequencing data of peripheral blood mononuclear cells. Thai J. Vet. Med.

[19] Dodt M, Roehr J.T, Ahmed R, Dieterich C (2012). FLEXBAR-flexible barcode and adapter processing for next-generation sequencing platforms. Biology (Basel).

[20] Dobin A, Davis C.A, Schlesinger F, Drenkow J, Zaleski C, Jha S, Batut P, Chaisson M, Gingeras T.R (2013). STAR:Ultrafast universal RNA-seq aligner. Bioinformatics.

[21] Wolf F.A, Angerer P, Theis F.J (2018). SCANPY:Large-scale single-cell gene expression data analysis. Genome Biol.

[22] Pedregosa F, Varoquaux G, Gramfort A, Michel V, Thirion B, Grisel O, Blondel M, Prettenhofer P, Weiss R, Dubourg V, Vanderplas J, Passos A, Cournapeau D, Brucher M, Perrot M, Duchesnay É (2011). Scikit-learn:Machine learning in python. J. Mach. Learn. Res.

[23] LeCun Y, Bengio Y, Hinton G (2015). Deep learning. Nature.

[24] Abadi M, Agarwal A, Barham P, Brevdo E, Chen Z, Citro C (2016). TensorFlow:Large-scale machine learning on heterogeneous systems.

[25] Liu J, Hill B.J, Darko S, Song K, Quigley M.F, Asher T.E, Morita Y, Greenaway H.Y, Venturi V, Douek D.C, Davenport M.P, Price D.A, Roederer M (2019). The peripheral differentiation of human natural killer T cells. Immunol. Cell. Biol.

[26] Wu L, Van Kaer L (2011). Natural killer T cells in health and disease. Front Biosci (Schol Ed).

